# Salivary characteristics and oral microbial dynamics in patients before and after maxillectomy with obturator prosthesis: a pilot prospective cohort study

**DOI:** 10.1007/s44445-026-00128-0

**Published:** 2026-03-07

**Authors:** Thabet Asbi, Yaniv Mayer, Rafael Sarikov, Jamil A. Shibli, Ilan Hirsh, Doron Haim, Amin Boukhari, Zvi Gutmacher

**Affiliations:** 1 Department of Periodontology, School of Graduate Dentistry, Rambam Health Care Campus, Haifa, Israel; 2 Research and Innovation Department, Maccabi-Dent, Tel-Aviv, Israel; 3 Guarulhos University, Guarulhos, Brazil; 4 Present Address: Technion - Israel Institute of Technology, Haifa, Israel; 5 Department of Prosthodontics & Maxillofacial Rehabilitation, School of Graduate Dentistry, Rambam Health Care Campus, Haifa, Israel; 6 Albert Einstein Faculty of Health Sciences, Albert Einstein Israelite Hospital, São Paulo, Brazil

**Keywords:** Maxillectomy, Obturator prosthesis, Microbiology, Saliva analysis, Oral rehabilitation

## Abstract

Maxillectomy produces extensive oral and maxillofacial defects that disrupt normal physiology and often require obturator prostheses for functional and aesthetic rehabilitation. Although these devices are routinely used, their influence on salivary characteristics and oral microbiota remains insufficiently understood. To compare pre- and postoperative salivary parameters, periodontal indices, and selected microbial species in patients undergoing partial maxillectomy and rehabilitated with obturator prostheses. In this pilot prospective cohort study, 14 adults scheduled for maxillectomy were evaluated. Unstimulated whole saliva was collected at baseline and at 6–8 months post maxillectomy. Clinical examinations assessed salivary flow, viscosity, periodontal indices, and semi-quantitative microbial profiles using commercial hybridization assays. Statistical tests were selected according to data type. Probing depth increased modestly (3.7 ± 0.7 mm to 3.9 ± 0.8 mm; *P* = 0.02), while plaque and gingival indices showed no significant changes. No significant changes were detected in either salivary flow and viscosity (6.97 ± 1.86 ml to 8.58 ± 2.29 ml, P = 0.58 and 1.50 ± 0.97 cps to 1.54 ± 0.96 cps, *P* = 0.64, respectively). Similarly, no statistically significant changes were detected in the prevalence of the selected microbial species, although a non-significant decrease in Aggregatibacter actinomycetemcomitans and an increase in Porphyromonas gingivalis were observed. Most other fungal and bacterial species showed similar prevalence across time points. Within the early postoperative period, salivary characteristics and oral microbial patterns remained generally unchanged following maxillectomy and obturator placement. Given the small and heterogeneous cohort, these preliminary outcomes warrant cautious interpretation and underscore the need for larger studies with extended follow-up time.

**Supplementary Information:**

The online version contains supplementary material available at 10.1007/s44445-026-00128-0.

## Introduction

Maxillectomy is performed to manage a range of conditions, including benign and malignant tumors, congenital anomalies, and traumatic injuries. Such procedures create extensive defects that directly affect speech, mastication, deglutition, and facial form. (Keyf 2001) The resulting communication between the oral cavity and nasal or sinus spaces exposes previously isolated anatomical regions to saliva, microorganisms, and environmental irritants. (Brown et al. 2000) Consequently, the defect due the maxillectomy, affects a patient’s quality of life, often leading to social isolation due to functional and aesthetic limitations. (Matsuyama et al. 2006) Therefore, the primary objective following maxillectomy is the rehabilitation of orofacial structures for restoring both function and aesthetics parameters. (Davison et al. 1998; Liu et al. 2006; Cordeiro and Santamaria 2000).

Two primary reconstruction approaches exist after maxillectomy procedures: One, using a free tissue flap with or without bone grafting with or without implants. And the other is removable obturator prosthesis, which requires manual removal by the patient for cleaning and maintenance. (Futran et al. 2002; Pigno 2001) The obturator serves as a protective barrier between the oral cavity and the surgical defect while simultaneously restoring facial contours, enhancing mastication and speech, and providing structural support for the lips and cheeks. (Rieger et al. 2002) An important advantage of obturator-based reconstruction is that it allows direct visualization of the maxillectomy site during routine follow-up visits, facilitating early detection of tumor recurrence or other postoperative complications. In addition, obturators often provide more predictable aesthetic outcomes than surgical reconstructions, which may be affected by scarring or soft-tissue distortion. They can also restore lost tissues immediately, and in many cases, replacement of missing teeth can be incorporated into the same prosthetic design. (Depprich et al. 2008).

Most obturators consist of a metal framework supporting an acrylic resin component that seals the maxillary defect. (Morgan and Wilson 2001; Engelhardt 1974) However, the acrylic surface may exhibit micro-irregularities created during polymerization that favor colonization by bacteria and fungi. (Engelhardt 1974; Glass et al. 2001; O'Reilly 2003; Zissis et al. 2006; Perezous et al. 2005; Sumi et al. 2002, 2003; Budtz-Jørgensen et al. 1996) Biofilm persistence on prosthetic materials can complicate hygiene efforts and may contribute to local or systemic infections, particularly in medically compromised or elderly individuals. (Costerton et al. 1999; Senpuku et al. 2003) Previous studies have demonstrated that prosthesis-associated biofilms can harbor pathogens linked to periodontal disease, denture stomatitis, and respiratory infections. (Sonis et al. 1978; Karpovich-Tate 2000; Milroy et al. 1989).

Despite the widespread clinical use of obturator prostheses, longitudinal data evaluating their impact on salivary characteristics and oral microbial ecology remain scarce. Therefore, this pilot prospective study was designed as a preliminary investigation to assess short-term changes in salivary parameters and the prevalence of selected oral microorganisms following maxillectomy and obturator rehabilitation.

## Materials and methods

### Study design

This pilot prospective observational cohort study was conducted between 2016 and 2020 and adhered to the ethical principles outlined in the Declaration of Helsinki. The study followed the Strengthening the Reporting of Observational Studies in Epidemiology (STROBE) guidelines. All participants were evaluated at two time points: prior to maxillectomy (baseline) and 6–8 months after surgery. The 6–8-month follow-up interval was selected to allow completion of surgical healing, stabilization of obturator use, and establishment of daily hygiene routines, while minimizing attrition in this medically complex population. This study was conducted at the Department of Prosthodontics and Maxillofacial Rehabilitation, Rambam Health Care Campus, Haifa, Israel.

As a pilot study, no formal sample size calculation was performed. The cohort size was determined by case availability during the study period.

### Ethical approval

The study protocol received approval from the Rambam Health Care Campus Ethics Committee (Approval ID: 0259–17-RMB; approved 24, August 2017). Written informed consent was obtained from all participants before enrollment.

### Patient selection

#### Inclusion criteria

- Adults aged ≥ 18 years.
- Scheduled for partial or total maxillectomy requiring obturator placement.
- Able to provide informed consent and willing to sign.
- Obturators supported by soft tissues or by a combination of teeth and soft tissues.

#### Exclusion criteria

- Improperly fitting or maladjusted obturators at follow-up.
- Physical limitations preventing adequate hygiene of the obturator or remaining dentition.
- Known allergy to materials used in obturator fabrication.
- Pregnancy or planned pregnancy.
- Use of systemic antibiotics or antifungal agents within 4 weeks before saliva collection.
- Diagnosed psychiatric conditions impairing cooperation.

### Surgical and prosthetic procedures

**Fig. 1:**
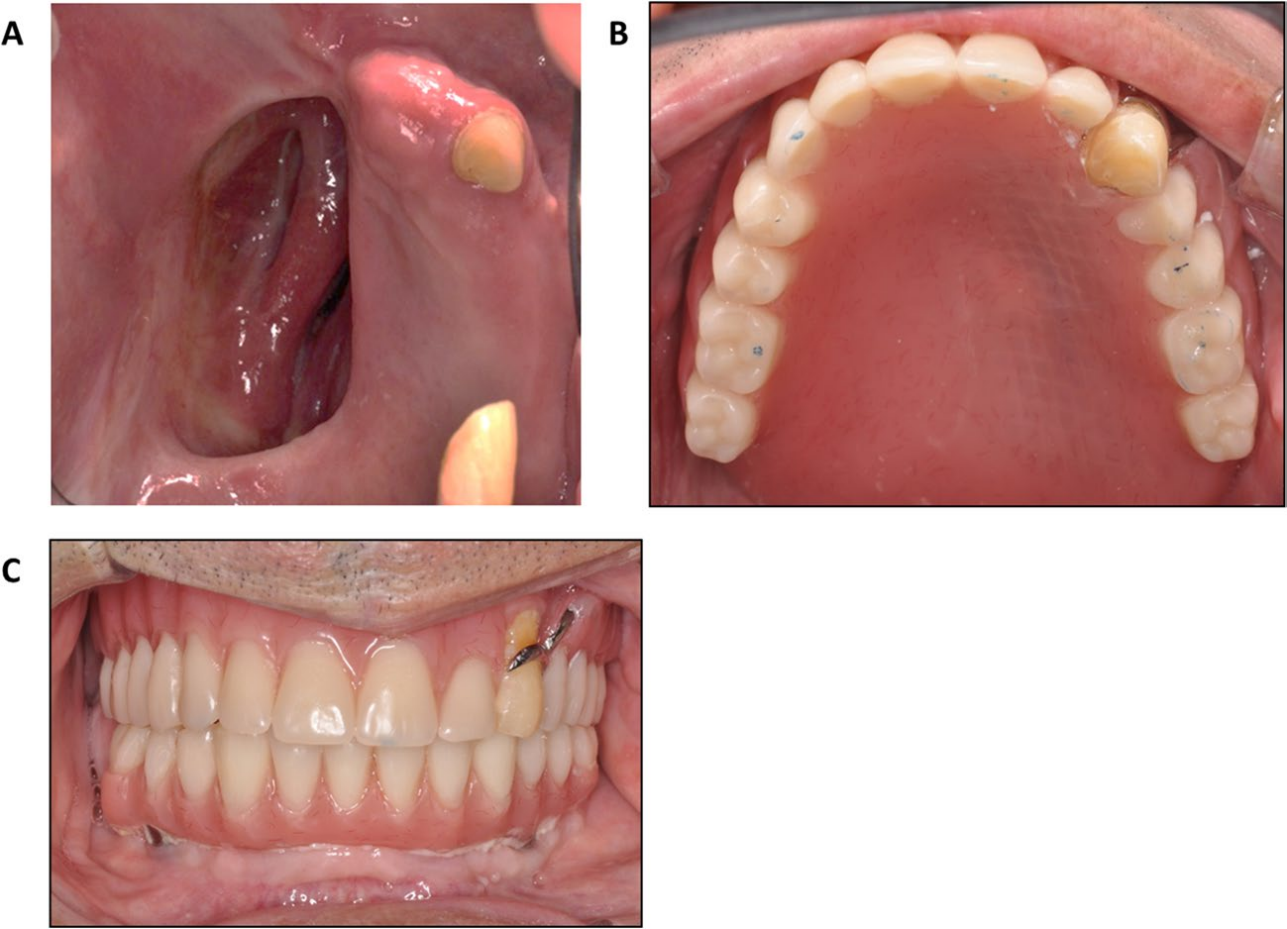
Representative clinical views of a resected hemimaxilla and corresponding obturator prosthesis eight months after maxillectomy. (**A**) Intraoral view demonstrating the healed hemimaxillectomy defect. (**B**) Occlusal view of the obturator prosthesis showing the tissue-surface adaptation and defect-sealing component. (**C**) Fold-to-fold view of the obturator illustrating extension, contour, and peripheral seal design

All maxillectomy procedures were performed by either Otorhinolaryngology or Oral and Maxillofacial Surgery specialists. Prior to the maxillectomy procedure, the maxillofacial prosthodontist fabricates the obturator. Immediately following resection, the maxillofacial prosthodontist adjusts the obturator to fit the defect. Designs consisted of a cast-metal framework with an acrylic resin extension to seal the defect and support soft tissues and prosthetic teeth when indicated Fig. 1.

### Clinical assessment procedures

#### Baseline visit (Preoperative)

Patients were screened, provided with detailed information about the study, and signed informed consent. At this visit, demographic and health-related information, including age, sex, general medical history, and smoking status was recorded for each participant. Participants also underwent:

- Extraoral and intraoral examination.
- Periodontal evaluation including plaque index (PI) (Silness and Löe 1964), gingival index (GI) (Löe and Silness 1963), and periodontal pocket depth (PPD).
- Collection of unstimulated whole saliva samples following standardized protocols.
- Oral hygiene reinforcement; periodontal treatment was provided when needed.

#### Follow-up visit (6–8 months postoperative)

At the second visit, patients underwent identical clinical and salivary evaluations to assess postoperative changes in oral health, salivary characteristics, and microbial composition.

### Saliva collection and analysis

#### Collection protocol

Unstimulated whole saliva was collected between 9:00–12:00 AM using the Navazesh method. (Lyons et al. 2023) Patients refrained from eating, drinking, or toothbrushing for at least one hour prior to sampling. Each participant sat upright with the head tilted slightly forward and expectorated into a sterile calibrated tube over five minutes. Samples were immediately labeled, stored at 4 °C, and transported for laboratory analysis.

#### Salivary parameters

- **Flow rate (mL/min):** calculated as total volume divided by collection time.
- **Viscosity:** measured in triplicate at 37 °C using an LVT Wells-Brookfield cone/plate viscometer. Instruments were calibrated before testing using manufacturer-supplied viscosity standards.

### Microbial assessment

#### Laboratory procedures

Saliva samples were analyzed at an accredited external laboratory (AMC-Medical Center Laboratories, Rishon-Lezion, Israel). A predefined pathogenic bacteria and fungi were detected using Micro-IDent® and Fungiplex® Candida hybridization assay kits (Hain Lifescience, Germany), performed according to manufacturer protocols.

The analyzed species included:

- **Periodontal pathogens:** A. actinomycetemcomitans, P. gingivalis, P. intermedia, T. forsythia, T. denticola.
- **Streptococcal species:** S. mutans, S. mitis, S. agalactiae.
- **Candida species:** C. albicans, C. tropicalis, C. krusei, C. glabrata.

The hybridization kits categorize results into semi-quantitative levels (e.g., low, moderate, high) based on predefined signal intensity thresholds. Results were reported as positive or negative and categorized semi-quantitatively according to kit standards.

#### Reproducibility analysis

Duplicate testing was conducted for each sample. The “n-fold difference” (ratio of repeated measures) quantified assay reproducibility, where a value of 1 indicated identical results and deviations indicated proportional variance. This ratio reflects analytical consistency, not microbial load table s1.

### Statistical analysis

Normality of continuous variables was assessed using the Shapiro–Wilk test. Continuous variables with normal distribution (salivary flow, viscosity, PPD) were analyzed using paired t-tests. Non-normally distributed variables (GI, PI, semi-quantitative microbial data) were evaluated using Wilcoxon or McNemar tests as appropriate. Microbial shifts across time points were examined using marginal homogeneity testing. Statistical analyses were performed using GraphPad Prism 9.0 (GraphPad Software, San Diego, CA). Significance was set at *p* < 0.05.

## Results

Fourteen patients from the Prosthodontic and Maxillofacial Rehabilitation Department in Rambam Hospital, Haifa, Israel, participated in this study. The cohort comprised 5 women and 9 men, with an age range of 32 to 77 years (mean age 63.42 ± 12.26). All patients had oral cavity tumors, underwent partial maxillectomy, and received an obturator (supported on teeth and soft tissue) immediately following the surgical procedure Table 1. At baseline, 10 patients were diagnosed with periodontitis and 4 were edentulous. The mean number of teeth present prior to maxillectomy was 12.93 ± 11.77 (range: 0–28) and following surgery the mean was 9.86 ± 9.13 (range: 0–22). A total of 46 teeth were extracted as part of the oncological treatment plan. Details of the periodontal and dental status of each participant are provided in Table 2. No postoperative complications, infections, or unusual clinical events were observed in any of the patients during the follow-up period. None of the participants required antibiotic treatment between the two time points. Furthermore, all patients were caries free.

**Table 1:** Demographic and clinical characteristics of the study cohort (*n* = 14)

Chemotherapy	Radiotherapy	Time until post op measurements (M)	Teeth After (nr.)	Teeth Before (nr.)	Periodontal Diagnosis	Type of surgery	Type of malignancy	Sex (Male (M)/Female(F))	Age	Patient number
No	Yes	6 M	22	28	STAGE 2 GRADE B	HEMIMAXILLECTOMY	AMELO	M	32	1
No	Yes	9 M	3	5	STAGE 4 GRADE C	HEMIMAXILLECTOMY	SCC	M	44	2
No	Yes	12 M	0	0	-	INFRASTRUCTURE-SUBTOTAL MAXILLECTOMY	ACC	M	56	3
No	No	7 M	12	23	STAGE 4 GRADE C	BILATERAL MAXILLECTOMY	SCC	M	57	4
No	Yes	8 M	0	0	-	PARTIAL MAXILLECTOMY	AMELO	M	63	5
No	Yes	6 M	16	19	STAGE 2 GRADE B	PARTIAL MAXILLECTOMY	SCC	M	66	6
No	Yes	9 M	9	9	STAGE 4 GRADE C	HEMIMAXILLECTOMY	ACC	F	66	7
No	Yes	6 M	25	26	STAGE 2 GRADE B	PARTIAL MAXILLECTOMY	ACC	F	66	8
Yes	No	10 M	14	28	STAGE 2 GRADE B	TOTAL MAXILLECTOMY	FIB OST	M	68	9
No	Yes	9 M	0	0	-	PARTIAL MAXILLECTOMY	SCC	F	70	10
No	Yes	9 M	1	1	STAGE 4 GRADE C	HEMIMAXILLECTOMY	BCC	M	71	11
Yes	Yes	8 M	18	24	LOC STAGE 3 GRADE C	MID-BILATERL MAXILLECTOMY	SCC	N	75	12
No	Yes	8 M	18	18	STAGE 4 GRADE C	HEMIMAXILLECTOMY	SCC	F	77	13
No	Yes	10 M	0	0	-	MID-BILATERAL MAXILLECTOMY	SCC	F	77	14

The table summarizes patient age, sex tumor type, surgical procedure, periodontal status, number f teeth before and after maxillectomy, exposure to chemotherapy and/or radiotherapy, and duration of follow-up prior to postoperative assessment. *SCC* Squamous Cell Carcinoma, *BCC* Basal Cell Carcinoma, *ACC Adenocystic Carcinoma, *AMELO* Ameloblastoma, *FIB OST* Fibroblastic Osteosarcoma, *M* male, *F* female

**Table 2:** Salivary microbiota results

Bacteria/Fungi	Pre-op N (%)	Post-op N (%)	*p*-value
C. albicans	5 (36%)	5 (36%)	1.00
C. tropicalis	1 (7%)	2 (14%)	1.00
C. krusei*	1 (7%)	0	-
C. glabrata	4 (29%)	3 (21%)	1.00
S. mitis	2 (14%)	2 (14%)	1.00
S. agalactiae	1 (7%)	2 (14%)	1.00
S. mutans	3 (21%)	4 (29%)	1.00
A. actinomycetemcomitans	3 (21%)	1 (7%)	0.50
P. gingivalis	2 (14%)	6 (43%)	0.13
P. intermedia	3 (21%)	2 (14%)	1.00
T. forsythia*	0	0	-
T. denticola	1 (7%)	4 (29%)	0.38

^*^Not applicable due to zero counts

Data are presented as the absolute number of patients positive for each microorganism and the corresponding percentage (%) out of the total study cohort (*n* = 14). The results are based on the semi-quantitative categorical cut-off values provided by the commercial hybridization kit

### Periodontal parameters

**Fig. 2:**
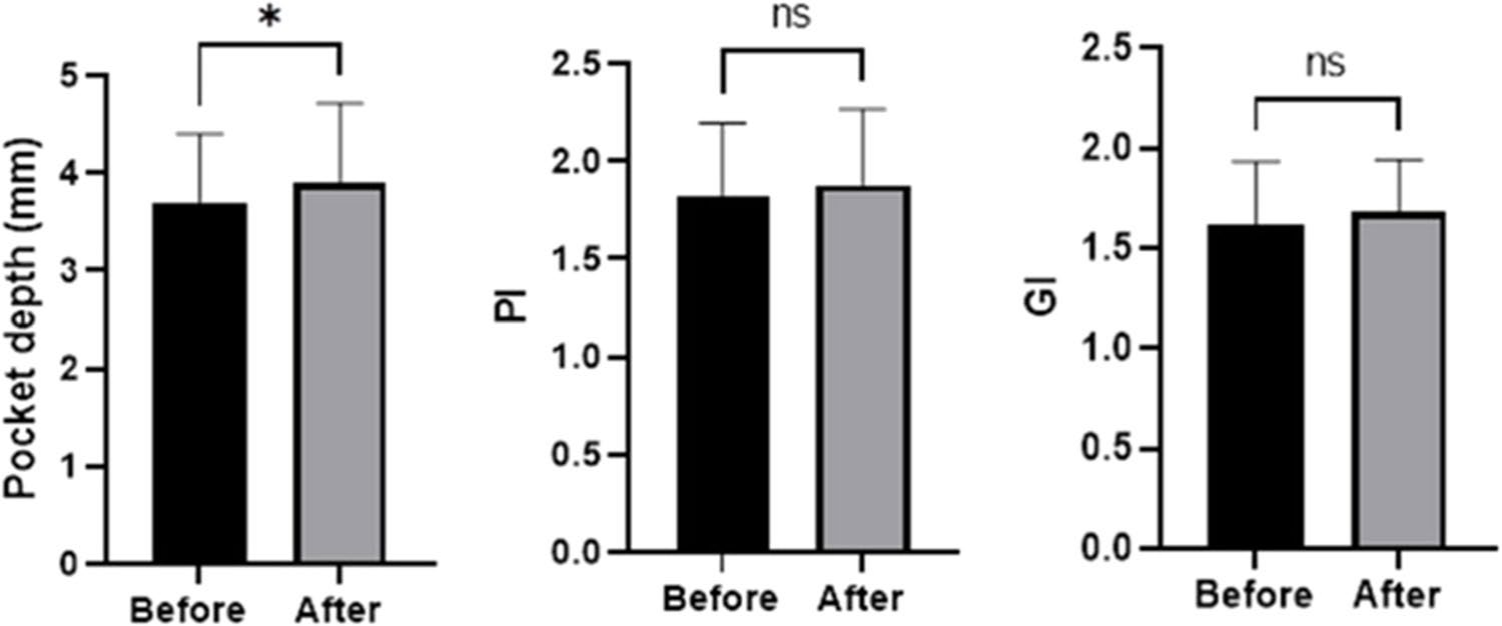
Clinical parameters showed a slight increase in periodontal pocket depth six months after obturator placement (*P* = 0.02, paired t-test), while PI and GI remained unchanged (*P* = 0.30, paired t-test, and *P* = 0.22, Wilcoxon test, respectively)

PPD increased slightly from 3.7 ± 0.7 mm to 3.9 ± 0.8 mm (*P* = 0.02), while PI and GI did not change (From 1.81 ± 0.38 to 1.88 ± 0.39, *P* = 0.30 and 1.61 ± 0.32 to 1.68 ± 0.26, *P* = 0.22, respectively) Fig. 2.

### Salivary measurements

**Fig. 3:**
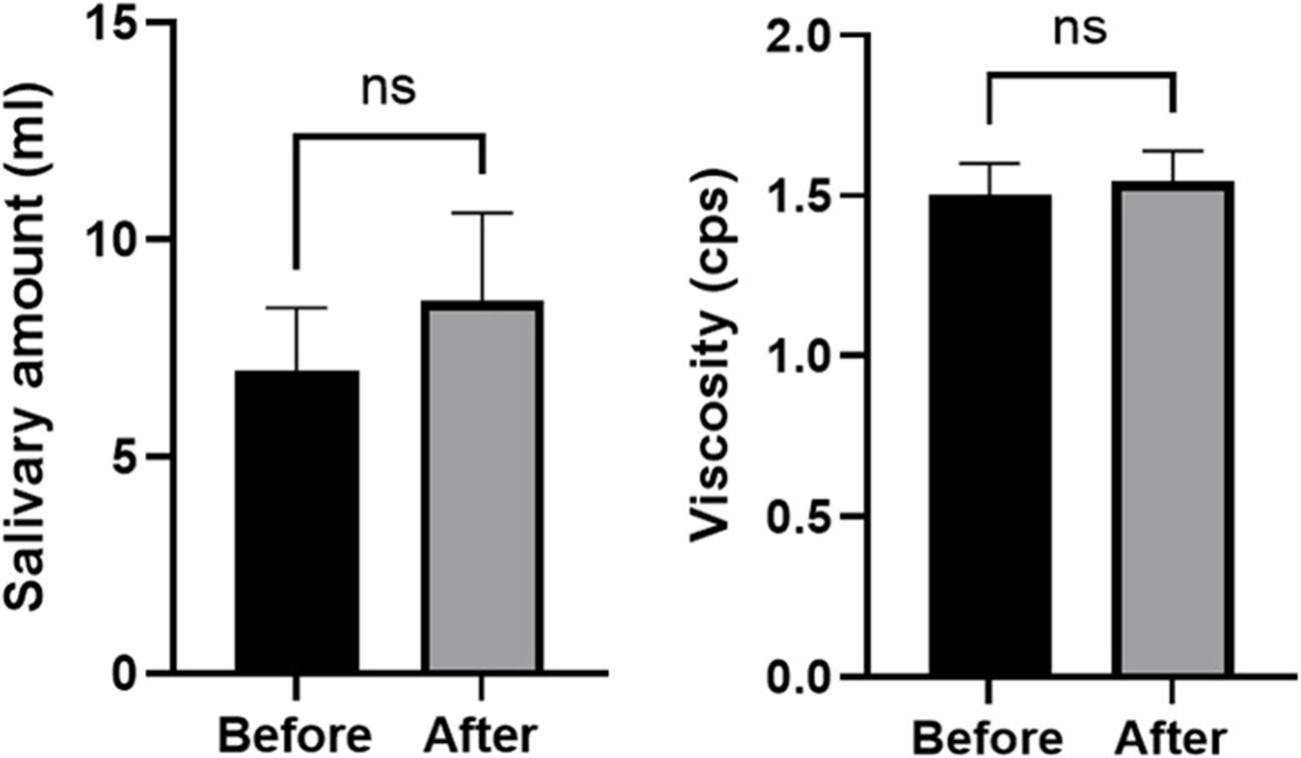
The salivary volume and viscosity remained unchanged six months after obturator placement. (*p* = 0.58 and *p* = 0.64, Wilcoxon test and paired t-test, respectively)

The average salivary volume was 6.971 ± 1.863 ml prior to maxillectomy and 8.584 ± 2.294 ml at the six-month follow-up; this was not statistically significant (*P* = 0.58). Salivary viscosity measurements demonstrated negligible variation between pre-operative and post-operative assessments, changing slightly from 1.5043 ± 0.967 cps to 1.5436 ± 0.958 cps, with no statistical significance (*P* = 0.64) Fig. 3.

### Microbial findings

**Fig. 4:**
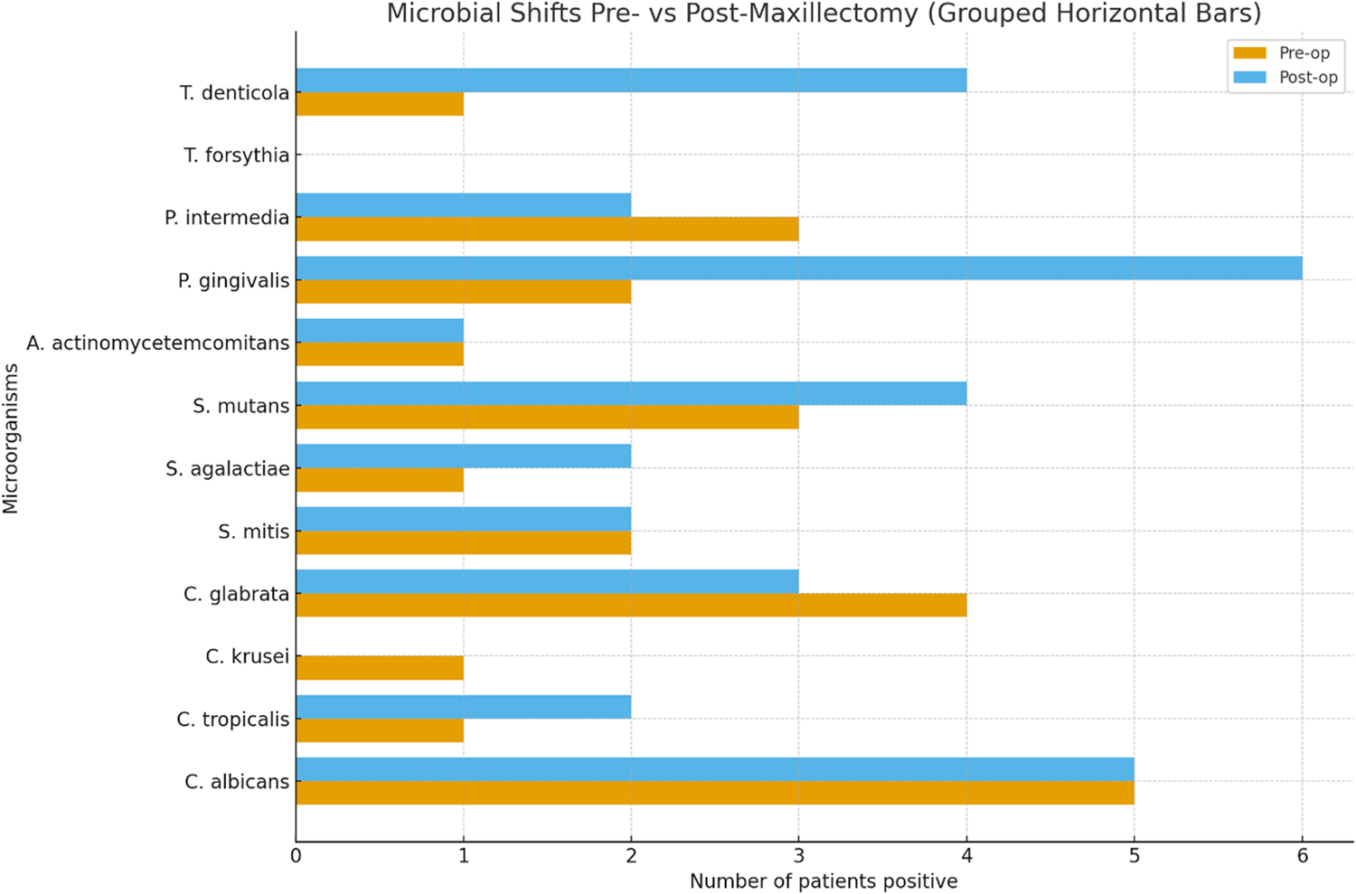
Microbial shifts before and after maxillectomy. Horizontal grouped bars show the number of patients positive for each microbial species pre-operatively and post-operatively. Data obtained using standardized hybridization assays (Micro-IDent® and Fungiplex® Candida). McNemar’s test was used for paired nominal comparisons

At the initial examination, C. albicans, C. glabrata, S. mutans, A. actinomycetemcomitans, and P. intermedia were detected, with no significant changes observed six months post-maxillectomy, except for a non-significant decrease in A. actinomycetemcomitans. Moderate levels of C. tropicalis, C. krusei, S. mitis, S. agalactiae, P. gingivalis, and T. denticola were also noted initially, with no changes by the study's conclusion, except for P. gingivalis, which showed a near-statistically significant increase, and C. krusei, which was undetected at the study's end. T. forsythia and C. Krusei were not detected at either time point. Table 2, Fig. 4. In addition to reporting the number and percentage of positive patients, Table s1 also presents the n-fold difference, which was calculated from duplicate analyses to assess the reproducibility of the hybridization assays. This parameter indicates the variation between repeated test runs (e.g., a value of 1 reflects identical results, whereas higher or lower values reflect proportional differences), and is not intended to represent absolute bacterial counts. The reproducibility values are shown to document the internal consistency of the assay and should be interpreted separately from the prevalence data.

## Discussion

This prospective pilot study evaluated the impact of maxillectomy combined with obturator rehabilitation on salivary characteristics, periodontal indices, and selected microbial species. Overall, salivary flow, viscosity, and most microbial findings remained stable during the first postoperative months, while a slight but significant increase in probing depth was noted.

### Interpretation of primary findings

The modest rise in probing depth without accompanying increases in plaque or gingival inflammation may suggest a localized alteration in periodontal support or challenges in cleaning around the surgical site and prosthesis. Similar observations have been reported previously, where anatomical modifications following maxillectomy influenced access for oral hygiene and altered natural self-cleansing mechanisms. (Depprich et al. 2008; Lyons et al. 2023) However, the stability of plaque and gingival indices suggests that patients in this cohort successfully maintained reasonable hygiene, likely reinforced by structured instructions and regular follow-up care. Nevertheless, given the observed trend toward increased P. gingivalis prevalence, clinicians should emphasize meticulous periodontal maintenance, individualized hygiene instruction, and periodic professional monitoring, particularly in patients with pre-existing periodontal disease or those receiving adjunctive oncologic therapies.

Reduced salivary output is frequently associated with radiotherapy or extensive head-and-neck surgery (Sonis et al. 1978), but recent evidence shows considerable variability depending on glandular-sparing techniques and individual patient factors. (Eisbruch et al. 1999; Kam et al. 2007) The maintained levels of unstimulated whole salivary flow suggest that obturator rehabilitation was not associated with measurable reductions in global salivary output during the early postoperative period in our cohort; nevertheless, these findings should be interpreted cautiously given the study’s limitations.

### Microbial dynamics and ecological considerations

Microbial analysis revealed no change across examined bacterial and fungal species. The reduction in A. actinomycetemcomitans, a pathogen associated with aggressive periodontitis (Nørskov-Lauritsen et al. 2019; Yang et al. 2004), may reflect improved accessibility for plaque removal or ecological changes introduced by the surgical defect. In contrast, an increase in the proportion of patients positive for P. gingivalis was observed; although this change did not reach statistical significance and the study was underpowered, the finding warrants cautious attention given the established role of P. gingivalis in periodontal disease. Recent genomic studies have shown that P. gingivalis exhibits remarkable adaptive capacity to colonize disrupted or prosthesis-associated environments (Murugaiyan et al. 2024).

The obturator's acrylic surface may contribute to these microbial patterns. Even minor surface irregularities can enhance microbial adhesion and biofilm maturation. (Depprich et al. 2008) Contemporary research confirms that prosthetic materials, particularly acrylic resins, harbor early and dense microbial colonization, with anaerobic pathogens showing a preferential affinity for rough or porous surfaces. (Mazurek-Popczyk et al. 2022) These mechanisms may help explain the observed rise in P. gingivalis following rehabilitation.

Interestingly, Candida species showed no meaningful postoperative increase. Earlier studies have reported elevated fungal colonization in patients receiving radiotherapy or long-term prosthesis use. (Pereira-Cenci et al. 2008; Davies et al. 2006) Current literature on denture-associated microbiomes also supports the concept that removable prostheses serve as distinct ecological niches. Reviews have demonstrated that denture patients often develop microbial communities that differ significantly from those of dentate individuals, with prosthesis material and hygiene practices exerting strong influences on microbial and fungal community structure. (Redfern et al. 2022; D'Ambrosio et al. 2023) Emerging evidence on 3D-printed acrylic materials also confirms their susceptibility to bacterial and fungal biofilm formation. (Mazurek-Popczyk et al. 2022; Melo 2020).

### Clinical implications

Within the limitations of the small and heterogeneous sample, obturator rehabilitation was not associated with statistically detectable shifts in the prevalence of the tested microorganisms during the early postoperative period; nevertheless, definitive conclusions regarding microbial balance cannot be drawn. Nonetheless, the observed trend toward increased P. gingivalis colonization highlights the importance of stringent periodontal maintenance, meticulous prosthesis hygiene, and frequent professional recall appointments.

Advances in prosthetic fabrication, such as digital design, additive manufacturing, and antimicrobial surface technologies, have shown potential in reducing microbial adhesion to obturator materials. (Mazurek-Popczyk et al. 2022; Melo 2020) Incorporating these developments may further enhance long-term outcomes and minimize biofilm-related complications in this population.

### Study limitations

This study has several limitations that must be taken into consideration when interpreting the findings. The small sample size, which reflects the rarity and complexity of maxillectomy cases, limits the statistical power and reduces the ability to generalize the results to broader populations. Additionally, the cohort was heterogeneous with respect to tumor pathology, extent of surgical resection, and use of adjunctive therapies, all of which may influence salivary and microbial outcomes and thereby introduce confounding effects. This heterogeneous nature of the cohort, especially with respect to radiotherapy exposure, constitutes a major limitation. As radiotherapy profoundly affects salivary gland function and the oral microbiome, combining irradiated and non-irradiated patients may have introduced confounding effects, potentially masking subgroup-specific biological responses and limiting attribution of findings to obturator rehabilitation alone. An additional limitation is the absence of a control group. Without comparison to alternative reconstructive approaches or to prosthesis wearers without maxillectomy, it is not possible to attribute the observed findings specifically to obturator rehabilitation. The single-arm design therefore limits causal inference and isolation of the obturator’s independent effect. Microbial assessment was based on semi-quantitative hybridization assays, which, although clinically useful, lack the depth and resolution of sequencing-based methods and were restricted to a selected panel of organisms; thus, broader microbiome alterations may have gone undetected. The relatively short follow-up period may also have limited the detection of microbial or clinical changes that evolve over longer durations of prosthesis wear and the 6–8-month follow-up period may be insufficient to capture longer-term ecological shifts or biofilm maturation on prosthetic surfaces. Finally, the study did not evaluate microbial exchange between the oral cavity and the nasal or sinus regions, despite the anatomical communication created by maxillectomy that may facilitate cross-site colonization. These limitations underscore the preliminary nature of the findings while highlighting the need for larger, longitudinal, and methodologically comprehensive future studies.

Future research should include larger multicenter cohorts, longer follow-up intervals, and high-resolution microbiome sequencing to better characterize microbial and fungal changes. Additional studies evaluating modern prosthetic materials, such as antimicrobial-coated resins and digitally fabricated obturators, are warranted given their promising early results. (Mazurek-Popczyk et al. 2022; Melo 2020) Furthermore, simultaneous assessment of nasal, sinus, and oral microbiota may help clarify cross-site colonization patterns created by maxillary defects (Albu and Roman 2025).

## Conclusions

This prospective pilot study provides preliminary data on salivary characteristics, periodontal parameters, and the prevalence of selected oral microorganisms following maxillectomy and obturator rehabilitation. Within the 6–8-month postoperative period, no statistically significant changes were detected in salivary flow or viscosity, and the prevalence of most tested microbial species remained stable, although a small increase in probing depth and a non-significant rise in Porphyromonas gingivalis prevalence were observed.

Future studies should include larger, multicenter cohorts stratified by radiotherapy exposure and surgical extent. Comparative investigations between conventional and digitally fabricated obturators may provide insight into material-related microbial dynamics. High-resolution methodologies such as 16S rRNA gene sequencing or shotgun metagenomics are recommended to characterize broader microbial community shifts beyond targeted pathogen panels.

## Supplementary Information

Below is the link to the electronic supplementary material.

Supplementary file1 (DOCX 15 KB)

## Data Availability

No datasets were generated or analysed during the current study.
