# Supplementary material for: Salivary characteristics and oral microbial dynamics in patients before and after maxillectomy with obturator prosthesis: a pilot prospective cohort study
Source: Saudi Dent J. 2026 Mar 7;38(3):25. doi: 10.1007/s44445-026-00128-0 (PMC12967776; doi:10.1007/s44445-026-00128-0)
Supplement: Supplementary file 1 — Supplementary file1 (DOCX 15 KB) [file 44445_2026_128_MOESM1_ESM.docx]

**Salivary Characteristics and Oral Microbial Dynamics in Patients Before and After Maxillectomy with Obturator Prosthesis: A Pilot Prospective Cohort Study**

**Supplementary Material**

Table S1 - The “N-fold difference” column represents reproducibility values from duplicate testing and does not indicate absolute bacterial counts.

| Bacteria / Fungi | Reproducibility Ratio (n-fold)* |
| --- | --- |
| C. albicans | 1 |
| C. tropicalis | 0.5 |
| C. krusei | 0 |
| C. glabrata | 1.33 |
| S. mitis | 1 |
| S. agalactiae | 0.5 |
| S. mutans | 0.75 |
| A. actinomycetemcomitans | 3 |
| P. gingivalis | 0.33 |
| P. intermedia | 1.5 |
| T. forsythia | 0 |
| T. denticola | 0.25 |
